# MXene-Chitosan Composites and Their Biomedical Potentials

**DOI:** 10.3390/mi13091383

**Published:** 2022-08-25

**Authors:** Parisa Iravani, Siavash Iravani, Rajender S. Varma

**Affiliations:** 1 School of Medicine, Isfahan University of Medical Sciences, Isfahan 81746-73461, Iran; 2 Faculty of Pharmacy and Pharmaceutical Sciences, Isfahan University of Medical Sciences, Isfahan 81746-73461, Iran; 3 Regional Centre of Advanced Technologies and Materials, Czech Advanced Technology and Research Institute, Palacký University in Olomouc, Šlechtitelů 27, 783 71 Olomouc, Czech Republic

**Keywords:** MXenes, chitosan, MXene-chitosan composites, biomedicine, MXene-based nanosystems

## Abstract

Today, MXenes with fascinating electronic, thermal, optical, and mechanical features have been broadly studied for biomedical applications, such as drug/gene delivery, photothermal/photodynamic therapy, antimicrobials/antivirals, sensing, tissue engineering, and regenerative medicine. In this context, various MXene-polymer composites have been designed to improve the characteristics such as physiological stability, sustained/controlled release behaviors, biodegradability, biocompatibility, selectivity/sensitivity, and functionality. Chitosan with advantages of ease of modification, biodegradability, antibacterial activities, non-toxicity, and biocompatibility can be considered as attractive materials for designing hybridized composites together with MXenes. These hybrid composites ought to be further explored for biomedical applications because of their unique properties such as high photothermal conversion efficiency, improved stability, selectivity/sensitivity, stimuli-responsiveness behaviors, and superior antibacterial features. These unique structural, functional, and biological attributes indicate that MXene-chitosan composites are attractive alternatives in biomedical engineering. However, several crucial aspects regarding the surface functionalization/modification, hybridization, nanotoxicological analyses, long-term biosafety assessments, biocompatibility, in vitro/in vivo evaluations, identification of optimization conditions, implementation of environmentally-benign synthesis techniques, and clinical translation studies are still need to be examined by researchers. Although very limited studies have revealed the great potentials of MXene-chitosan hybrids in biomedicine, the next steps should be toward the extensive research and detailed analyses in optimizing their properties and improving their functionality with a clinical and industrial outlook. Herein, recent developments in the use of MXene-chitosan composites with biomedical potentials are deliberated, with a focus on important challenges and future perspectives. In view of the fascinating properties and multifunctionality of MXene-chitosan composites, these hybrid materials can open significant new opportunities in the future for bio- and nano-medicine arena.

## 1. Introduction

MXenes and their derivatives have been widely explored in the field of supercapacitors [1], sensors [2], energy storage [3], diagnostics [4], (photo)catalysis [5], and drug delivery [6,7,8,9] due to their special properties such as large surface area, superior near-infrared (NIR) responsiveness, excellent mechanical strength, rich surface chemistry, exceptional hydrophilicity, and easy of surface functionalization/modification [10,11,12,13,14]. These materials exhibited several advantages such as broadband absorption, light-harvesting features in the NIR region, strong light-to-heat conversion capabilities, metallic conductivity, biocompatibility, biodegradability, significantly negative zeta potential, and abundant surface functional groups [4,15]. In this context, composites of MXenes and polymers have been designed with fascinating physicochemical properties for biomedical applications. To improve the physiological stability, sustained/controlled drug release behaviors, drug loading capacity [16], biodegradability, biocompatibility [17], and targeting properties, several MXene-polymer (nano)composites have been designed [18,19,20,21,22,23]. Polymer-functionalized MXene composites exhibited enhanced the physiological stability, stimuli-responsiveness [24], high sensitivity/selectivity [25], improved biocompatibility [26], and contrast enhancement, introducing them as promising alternatives in bio- and nano-medicine [15,27,28,29,30,31]. Multifunctional MXene-based (nano)composites have shown suitable applicability for high-performance energy-related devices and flexible bioelectronics [32,33,34]; they also exhibited useful photocatalytic performances, electromagnetic interference (EMI) shielding, and high charge storage [15,31,35].

Overall, MXenes have been fabricated through the selective removal of “A” layer from their MAX or non-MAX phase parents by acid etching, where A is mostly group 13 or group 14 elements in the periodic table [13,36]. A variety of top-down and bottom-up strategies have been reported for the synthesis of MXenes and their derivatives such as the urea glass technique [37], chemical vapor deposition [38], molten salt etching [39], hydrothermal synthesis [40], electrochemical fabrication [41], and bioinspired/biomimetic methods [23]. Among them, chemical vapor deposition and wet etching methods are widely introduced for synthesizing MXenes [42]. Notably, the assortment of proper optimization conditions and synthesis methods highly depends on their MAX precursors. Besides, high-quality MXenes with the presence of terminations could be produced through the application of various wet etching techniques, generating MXenes with basically hydrophilic nature [43]. For the synthesis of chitosan/MXene hybrid composites, there are some reports as exemplified by chitosan/MXene alternating layered composites which could be synthesized by applying layer by layer assembly technique that is inspired by the electrostatic interaction between an oppositely charged chitosan solution and MXene slurry [44]. In another study, MXenes (Ti_3_C_2_T_x_) were introduced to chitosan-based porous carbon microsphere to produce sandwich-like structures via the electrostatic interaction [45]. MXenes with typical formula of M_n+1_X_n_T_x_ exhibited alluring capabilities for the surface amendment; they can be further functionalized/modified with a variety of biocompatible/bioactive agents, therapeutic drugs, photosensitizers, and immune adjuvants due to the presence of functionalities such as -O, –F, and –OH, hydrophilicity, and high surface area [46].

Chitosan with biodegradability, non/low toxic effects, and renewability can be applied for constructing novel MXene-chitosan composites with biomedical applicability [47]. The application of chitosan can also improve the mechanical properties of MXenes [48]. For instance, chitosan-reinforced MXene (Ti_3_C_2_X) films were prepared with shell-like nano-laminar microstructures. As a result, the tensile strength of these MXene-based films was improved from 8.20 to 43.52 MPa, increasing 5.3 times. In addition, the electrical resistivity of them were enhanced from 0.39 (0 wt%) to 54.91 mΩ cm (14 wt%) [48]. On the other hand, MXene-chitosan composites have been applied for constructing EMI shielding materials such as MXene/chitosan-derived hybrid carbon aerogels with hierarchical pore structures for durable EMI shielding [49]. When MXenes and chitosan were hybridized, excellent electrical conductivity and EMI shielding properties can be obtained, providing great opportunities for designing next-generation EMI shielding materials with biomedical potentials [47]. For instance, MXene/chitosan/silver nanowire sandwich films were constructed through a vacuum-assisted filtration technique, with electrical conductivity of 11,459.1 S/m [50]. Also, Tan et al. [44] have introduced chitosan/MXene multilayered films with EMI shielding applicability and excellent thermal conductivity (6.3 W m^−1^ K^−1^), which can be further explored for manufacturing next-generation devices. Herein, recent developments in the use of MXene-chitosan composites with applications in biomedicine such as sensing [51], antimicrobials [52], photothermal therapy [53], drug delivery [54], and cancer therapy [55,56] are covered (Table 1), focusing on important challenges and future perspectives.

## 2. MXene-Chitosan Composites 

### 2.1. Sensing

**Figure 1:**
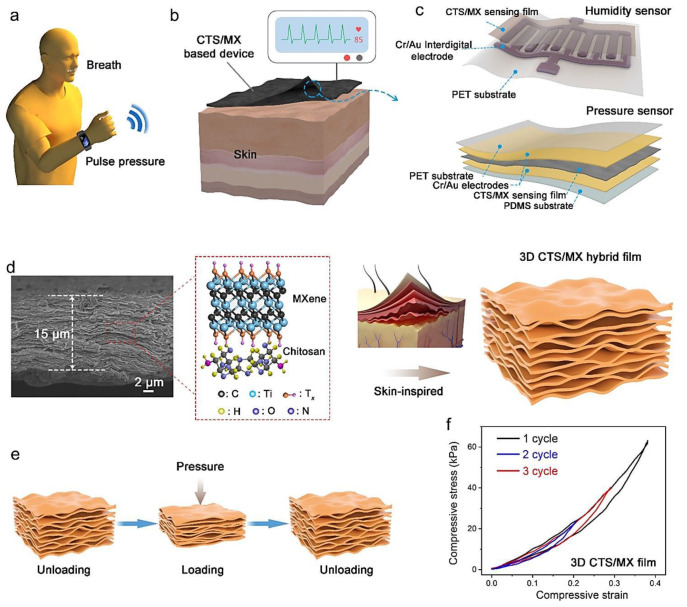
(**a**–**d**) The preparative process of biocompatible chitosan (CTS)/MXene (MX) hybrid film and the design of flexible bimodal humidity and pressure sensor for human health detection purposes. (**e**) The sensing mechanism of the designed sensor for the detection of pressure. (**f**) Compressive stress-strain curves of the prepared hybrid film under various strain values. Adapted from Ref. [59] with permission. Copyright 2021 American Chemical Society.

MXene-based (nano)structures with outstanding electrical and optical features have been widely explored for sensing applications [60]. However, very limited studies have focused on the biosensing applications of MXene-chitosan hybrid composites with different properties. Hroncekova et al. [51] reported the synthesis of MXene (Ti_3_C_2_T_X_)-chitosan nanocomposites to design an amperometric biosensor for the specific detection of a potential prostate cancer marker (sarcosine) in urine samples. Accordingly, the low limit of detection (LOD) was ~18 nM and linear range was up to ~7.8 µM (the response time was ~2 s) [51]. These MXene-chitosan composites need to be further explored as potential materials in designing novel electrochemical biosensing platforms for clinical and biomedical diagnostics [61]. Additionally, MXenes are recognized as ideal materials for sensitive wearable strain sensors due to their special benefits of hydrophilicity, conductivity, and mechanical features. But still the unnecessary accumulation of MXene nanosheets during the synthesis process limited the transmission of electrons and reduced the conductivity; also it could reduce the mechanical potentials and sensitivity of sensors [61]. To overcome this challenge, conductive polyacrylamide hydrogels that were enabled by dispersion-enhanced MXene-chitosan hybrid assembly were prepared to design sensors with high sensitivity. These hybrid composites exhibited excellent conductivity along with mechanical strength and flexibility. They can be applied for designing platforms with self-adhesion properties and antibacterial performances. Future studies should be moved toward the construction of next-generation intelligent devices with broad applications in electronic skin and human motion detection [61]. Wang et al. [59] introduced flexible bimodal electronic skins for the detection of pressure (LOD = 3 Pa, stability > 3500 times, and response time of 143 ms) and humidity (stability > 20 days). These devices were constructed from biocompatible MXene-chitosan film (the kernel sensing layer) (Figure 1). These kinds of bifunctional sensors can be applied for the sensitive detection and discrimination of electrophysiological signals such as recognition of voice, finger bending, and human pulses along with the biochemical molecules (respiratory rate), providing next-generation multifunctional sensing devices for health and biomedicine applications [59]. 

**Figure 2:**
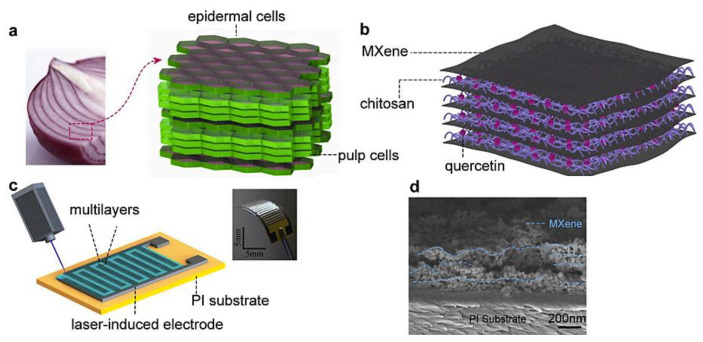
(**a**–**c**) The preparative process of onion-inspired MXene/chitosan-quercetin multilayers with their schematic structures for the designing of the flexible humidity sensor based on laser-induced interdigitated electrode upon polyimide (PI) substrate. (**d**) Cross-sectional scanning electron microscopy (SEM) that was obtained from the composites. Adapted from Ref. [64] with permission. Copyright 2020 Elsevier.

MXenes and their derivatives have shown great potential in constructing sensitive electrochemical biosensors [62]. An electrochemical sensor was constructed from multi-walled carbon nanotubes, MXene (Ti_3_C_2_), and chitosan for the detection of ifosfamide, acetaminophen, domperidone, and sumatriptan [63]. The prepared electrode exhibited improved electrocatalytic performances toward the oxidation of target analytes. In addition, the application of MXene with large surface area improved the conductivity and catalytic properties of the composites and could help in improving the LOD of targets along with the selectivity and reproducibility. According, ifosfamide, acetaminophen, domperidone, and sumatriptan were detected in the concentration ranges 0.0011–1.0, 0.0042–7.1, 0.0046–7.3, and 0.0033–61 μM with LOD of 0.00031, 0.00028, 0.00034, and 0.00042 μM, respectively. This sensor could be applied for voltametric monitoring of target analytes in urine and blood serum samples (the recoveries = > 95.21%) [63]. On the other hand, MXenes with advantages of hydrophilicity, tunable conductivity, and large surface area can be considered as promising candidates for the sensing of humidity and non-invasive monitoring of physiological events (e.g., respiration) [64]. In one study, onion-inspired assembling of MXene (Ti_3_C_2_T_x_) and chitosan-quercetin hybrid layer-by-layer was reported for the precise tracking of human breath (Figure 2). These hybrid structures could respond to H_2_O molecules. Since the chitosan-quercetin altered multilayers suppressed the environmental degradation of MXenes, providing an excellent and ultrafast response (317% at 90% RH, 0.75 s) with long-term stability (>15 days) [64]. These composites should be further evaluated for wearable human respiration monitoring with high accuracy, providing simple and feasible strategies for multipurpose physiological monitoring based on humidity sensing.

An enzyme-free biosensor with excellent anti-interference potential and reproductivity was designed utilizing MXene/chitosan/Cu_2_O electrode (as a biomimetic electrocatalyst) for the specific sensing of glucose and cholesterol with clinical diagnostic potentials [58]. Accordingly, the sensitivity for the detection of glucose was 60.295 µA·L/(mmol·cm^2^) with LOD of 52.4 µmol L^−1^, while the sensitivity for cholesterol detection was up to 215.71 µA·L/(mmol·cm^2^) with LOD low to 49.8 µmol L^−1^. They can be applied for analyzing multiple metabolites to overcome the disadvantages of an enzyme-based biosensor, which can pave the way for designing portable electrochemical devices with capabilities of sensing blood metabolites [58].

### 2.2. Antimicrobials 

MXenes have shown excellent antimicrobial effects against pathogenic bacteria through the physical damages, photocatalytic inactivation, and photothermal effects [65]; their antimicrobial activities were dose-dependent and higher than in the case of graphene-based materials [66]. MXenes with negatively charged surfaces and hydrophilicity illustrated efficient bacterial contact, causing bacterial inactivation with direct contact-killing mechanisms [67,68,69,70,71,72]; hydrogen bonding between oxygenate groups of MXenes and lipopolysaccharide strings of the bacterial cell membranes can be one of the important reasons for the inhibition of pathogenic bacteria by avoiding nutrient intake. However, the related interactions between these structures and bacterial cell membranes ought to be studied in detail [66]. In one study, encapsulated delaminated MXene (Ti_3_C_2_T_z_) flakes within chitosan nanofibers were constructed using an electrospinning technique [73]. These biocompatible hybrid nanofibers were employed in passive antibacterial wound dressing purposes. Accordingly, they exhibited suitable antibacterial effects against *Escherichia coli* (~95% reduction in colony forming units) and *Staphylococcus aureus* (~62% reduction in colony forming units) after 4 h of treatment. The direct mechanical destruction of bacterial cell membranes via MXene flakes was described as one the major ways of their antibacterial effects. Furthermore, these composites with hydrophilicity and negatively-charged flake surfaces owing to the reactive –O, –OH, and –F surface terminations could stimulate the bacterial agglomeration [73]. Wang et al. [52] utilized a poly l-lactic acid membrane for the assembling of positively-charged chitosan and negatively-charged silver-MXene on the surface via a layer-by-layer technique. The composite demonstrated an excellent growth inhibition ratio *E. coli* (91.27%) and *S. aureus* (96.11%) under 808 nm near-infrared laser radiation with synergistic photothermal antibacterial effects. Notably, this composite exhibited enhanced biocompatibility compared with the examined poly L-lactic acid membrane, which ought to be further explored as biomedical materials [52].

### 2.3. Drug Delivery and Cancer Therapy 

**Figure 3:**
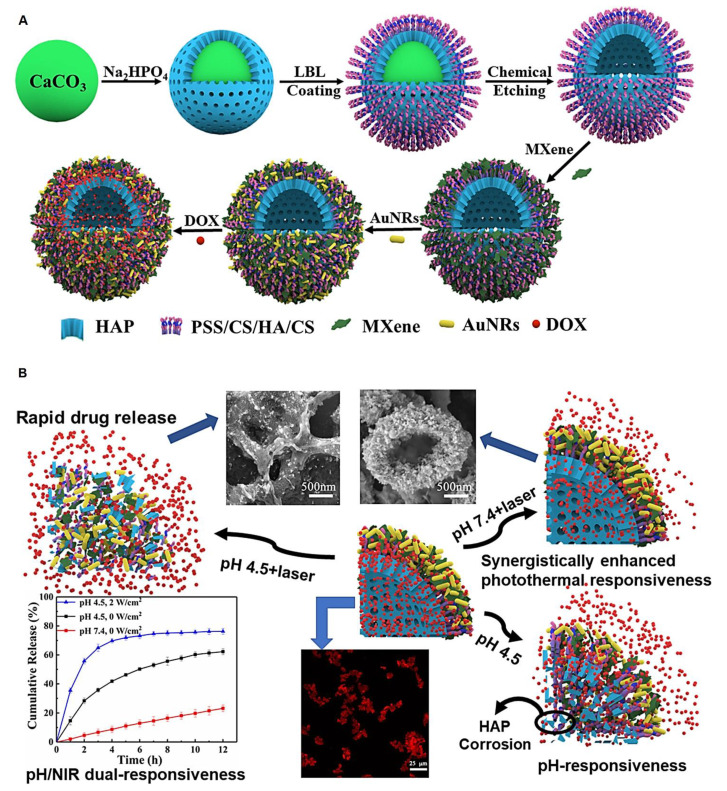
(**A**) The preparative process of drug delivery microcapsules that were constructed from hollow hydroxyapatite (HAP), chitosan (CS), hyaluronic acid (HA), gold nanorods (Au NRs), and MXene. (**B**) These microcapsules with pH-/NIR-responsive drug release behavior were deployed for the targeted delivery of doxorubicin (DOX). Adapted from Ref. [54] with permission. Copyright 2021 Elsevier.

MXene-based systems have been designed with photo-/magnetic-responsive drug delivery potentials for chronic wound healing [74]. Furthermore, innovatively designed MXene-based delivery platforms were introduced with NIR laser-triggered and pH-responsive drug release behaviors for cancer therapy. Notably, surface-functionalized MXene-based drug delivery systems exhibited high drug-loading capacity, sustained/controlled release, and specificity/selectivity [55,56]. A pH/NIR multi-responsive microcapsule was constructed from hollow hydroxyapatite, chitosan, hyaluronic acid, gold (Au) nanorods, and MXene (Ti_3_C_2_) through a layer-by-layer technique for the targeted delivery of an anticancer drug (doxorubicin) [54]. The application of MXenes and Au nanorods could significantly enhance the photothermal conversion efficiency of this microcapsule, showing outstanding pH-/NIR-responsive drug delivery features and high drug loading efficiency along with suitable biocompatibility and controlled release behavior (Figure 3) [54]. 

### 2.4. Photothermal Therapy 

MXenes have shown excellent photothermal conversion efficiency, which make them suitable candidates for photothermal therapy and solar energy [75]. Several MXene-based structures have been constructed with photo-physical features for targeted cancer photothermal therapy [76]. Besides, MXene-based structures (e.g., muscle-inspired MXene/polyvinyl alcohol hydrogels) with outstanding mechanical features exhibited local hyperthermia of infected sites under NIR laser irradiation (808 nm) [77]. These materials with photothermal effects demonstrated broad-spectrum antibacterial performances against pathogenic bacteria along with the effective promotion of cellular proliferation, providing efficient nanoplatforms for inhibiting wound infections, and stimulating skin wounds healing [77]. MXene/quaternary chitosan membranes with mechanical robustness, excellent antioxidant activity, and tailored electronic conductivity were constructed in a bio-inspired by the “brick and mortar” structure of natural nacre for photothermal conversion with high efficiency [53]. These membranes exhibited significant tensile strength (50.93 MPa) with a Young’s modulus of 4.4 GPa due to the electrostatic interaction and hydrogen bonding between the nanosheets of MXenes and molecular chains of chitosan. Notably, the electronic conductivity could be adjusted by changing the weight ratio of MXene/quaternary chitosan, obtaining a maximum value of 128 S m^−1^; the antioxidant nature of quaternary chitosan contributed to significant radical scavenging capacity (>80%). These membranes with efficient photothermal conversion demonstrated great potentials in the field of photothermal therapy [53].

## 3. Biosafety Issues 

Biocompatibility and toxicity (toxicological and cytotoxicity properties) are two important aspects, which ought to be systematically analyzed for successful clinical translation of MXene-based composites with biomedical potentials [78,79,80,81,82]. The potential cytotoxic effects of these materials on human cells are chiefly associated with their physicochemical properties, cellular interactions, and accumulations in the targeted organs/tissues [83]. Thus, cellular/molecular interactions and toxicological aspects of these composites should be deeply investigated, including penetration/attachment, endocytosis, ROS, possible DNA damages, inflammatory reactions, apoptosis, etc. [84,85,86,87]. In some studies, physical damages, modifications in the subcellular internalization mechanisms, and the oxidative stress that is caused by the generation of active reactive oxygen species have been reported as possible toxicity mechanisms of MXene-based materials [88]. It appears that comprehensive and specific in vitro/in vivo studies are still required for delineation of toxicity mechanisms as well as long-term biosafety assessments. Some studies revealed that MXenes could have possible toxic effects on zebrafish embryo models (an in vivo study) [89]. The MXenes were up-taken by the zebrafish embryos with the highest NOEC (no observed effect concentration) of ~50 μg mL^−1^, the lethal concentration 50 of ~257.46 μg mL^−1^, and LOEC (lowest observed effect concentration) of ~100 μg mL^−1^. The toxicity of MXenes was dose-dependent and could be changed by altering the concentrations; no noticeable teratogenic effects were identified on the studied models at 100 μg mL^−1^. Notably, further neurotoxicity assessments illustrated that MXene-based structures had no meaningful toxic effects on neuromuscular activities at 50 μg mL^−1^. They can be classified as practically non-toxic materials at concentrations below 100 μg mL^−1^, based on the Acute Toxicity Rating Scale (ATRS) by the Fish and Wildlife Service [89]. Besides, the teratogenic phenotype analyses demonstrated that some MXene-based composites including Au/MXene and Au/Fe_3_O_4_/MXene had no acute toxic or teratogenic effects on zebrafish embryos at all the evaluated concentrations [90]. 

Pan et al. [91] introduced MXene-based composites for osteosarcoma phototherapy and enhanced tissue reconstruction. The results of in vivo toxicity assessments after 24 weeks upon implantation as well as the hematological and histological analyses illustrated no noticeable changes in the values compared to the control samples, showing non/low toxicity of these materials [91]. Besides, acute toxicity assessment of MXene-based composites was reported upon intravenous administration of these materials at 6.25, 12.5, 25, and 50 mg kg^−1^ [84]. Accordingly, the histocompatibility of the mice organs upon days 1 and 7 exhibited no evidence of pathologies and significant histomorphological alterations in the evaluated organs compared to the control samples, showing no acute toxicity and adverse effects from these composites. It was also indicated that the excretion with urine and feces was ~18.70% and ~10.35% after 48 h, respectively [84]. In another study, biocompatibility/biosafety assessments (in vivo) of MXene-based composites after single-dose intravenous administration at 5, 10, and 20 mg kg^−1^ to healthy lab mice demonstrated no noticeable toxicity and all the major vital signs were normal upon the 30-day observation period, with barely any deviation from the control; biochemical blood assays and the target organs examinations indicated no signs of toxic effects [92].

In addition, biocompatibility, pharmacokinetics, and biodegradability of these materials can be improved by employing eco-friendly synthesis techniques, hybridization with natural polymers (e.g., chitosan), and surface functionalization/modification with bioactive/biocompatible agents [66,89,92,93,94,95,96]. For instance, Pu et al. [97] utilized chitosan with renewability and non-toxicity advantages for fabricating nitrogen-doped MXene nanomaterials through an eco-friendly technique. These above-mentioned aspects can also improve their targeting features (selectivity and specificity), and also reduce possible off-target effects and undesired events such as aggregation or accumulation, which can hinder their future biomedical and clinical applications and reduce their functionality [12,82,98]. 

## 4. Conclusions and Future Outlooks

MXenes have been investigated in biomedical sciences due to their special thermal, electronic, optical, mechanical, and biological characteristics. These materials with the abundant surface functional groups can be simply functionalized or modified with a variety of polymers. Several MXene-polymer hybrid composites have been constructed with advantages of enhanced photothermal conversion efficiency, higher antibacterial activities, sensitivity/selectivity, contrast enhancement, and stimuli-responsiveness behaviors. Despite these benefits, there are still some important challenges regarding large-scale production, stability, storage, in vivo retention, and long-term biosafety, which can hinder the widespread applications of these materials at medical levels. Natural polymers such as cellulose and chitosan have been studied for designing hybrid MXene-based composites with improved biomedical potential and multifunctionality as well as reduced toxicity. Notably, finding suitable environmentally-benign techniques for the synthesis of MXenes and their derivatives ought to be further explored, focusing on optimization conditions, physiological stability, up-scalable production, surface chemistry characterization, nano-/eco-toxicological studies, long-term biocompatibility assessments, and pre-/clinical analyses. By adjusting interlayer spacing, surface functional groups/terminations, and synthesis/reaction conditions (such as pH or temperature), their optical, mechanical, electronic, and thermal properties can be further amended.

## Figures and Tables

**Table 1:** Some selected examples of MXene-chitosan composites and their applications.

MXene/Chitosan Composites	Applications	Advantages/Properties	Refs.
MXene (Ti_3_C_2_T_X_)-chitosan nanocomposites	(Bio)sensing	Ultrasensitive detection of prostate cancer biomarker; short response time (~2 s) and significant recovery index (~102.6%) for detecting sarcosine spiked into urine samples in a clinically relevant range	[51]
Multilayer MXene (Ti_3_C_2_)/chitosan/silver coatings	Antibacterial effects	Excellent antibacterial effects against Gram-negative bacteria (*Pseudomonas aeruginosa*) with reduction of ~99.97% and Gram-positive bacteria (*Staphylococcus aureus*) with reduction of ~88.9%.	[57]
MXene/chitosan/Cu_2_O electrode	(Bio)sensing	Superb sensing potentials for the detection of glucose and cholesterol, with preferable linear ranges covering the full concentration range in clinical diagnosis.	[58]
MXene/chitosan films	Real-time pulse and respiratory rate monitoring	High biocompatibility and flexibility	[59]
MXene/quaternary chitosan membranes	Photothermal therapy	Excellent mechanical robustness, high antioxidant performance, tailored electronic conductivity; high-performance photothermal conversion	[53]

## Data Availability

Not applicable.
